# Supplementary material for: Are carbon emissions trading and green financial instruments synergistic? -Comprehensive quantitative research based on content analysis
Source: PLoS One. 2024 Mar 7;19(3):e0298601. doi: 10.1371/journal.pone.0298601 (PMC10919601; doi:10.1371/journal.pone.0298601)
Supplement: S1 Data — (ZIP) [file pone.0298601.s001.zip › Data/地级市Z_F工作报告.zip!/╣π╢1⁄2/╔╟╬▓╩╨/└┤╫╘╣1⁄2╓┌║┼í╛┬φ┐╦╩2╛▌═°í┐_2008.docx]

各位代表：

我代表市人民政府向大会作政府工作报告，请予审议，并请政协各位委员和其他列席人员提出意见。

2007年工作回顾

　　2007年，市政府在省委、省政府和市委的正确领导下，在市人大及其常委会和市政协的监督支持下，坚持以邓小平理论和"三个代表"重要思想为指导，以科学发展观统领经济社会发展全局，认真贯彻党的十七大、省十次党代会、粤东会议精神，紧紧围绕市五次党代会提出的"抢抓新机遇，推进新跨越，共建新汕尾，努力打造活力汕尾、人文汕尾、和谐汕尾、清新汕尾"的奋斗目标，一手抓经济发展，一手抓社会稳定，促进了国民经济持续、快速、健康发展，顺利完成了市五届人大一次会议确定的各项任务，经济社会建设取得了新的成就。主要体现在"十个新"。

1、国民经济跃上新台阶。2007年全市完成生产总值295.38亿元，比上年增长17.1%，人均生产总值超过万元。工业总产值456.19亿元，增长25.7%，其中，规模以上工业完成总产值199.28亿元，增长38.1%。全社会固定资产投资175.81亿元，增长30.8%。外贸出口11.23亿美元，增长31.5%。财政一般预算收入12.16亿元，增长31.2%。社会消费品零售总额187.11亿元，增长21.3%。农业总产值109.20亿元，增长6.2%，水产养殖面积达3.28万公顷。三次产业结构进一步优化，由上年的22.6：42.5：34.9调整为20.8：44.0：35.2。市区居民人均可支配收入9567元，人均消费支出7722元，分别增长8.5%和4.7%。农民人均纯收入4273元，增长6.5%。万元GDP能耗控制在0.534/吨标煤以内。

2、重点项目建设取得新进展。2007年市重点建设项目27个，完成投资38亿元。信利TFT-LCD项目首条生产线已建成投产；德昌电子三期工程建设进展顺利；汕尾发电厂一期1、2号机组已并网发电；陆丰核电站永久性进厂道路已动工建设；海丰核电项目前期工作正在抓紧推进；甲东风电一期已投产；海丰华城石化储运生产项目即将投产；环品清湖新区开发不断推进；万福、碧桂园房地产项目加紧建设；市区污水处理厂首期工程已建成投入运营；市技校扩建工程即将竣工投入使用；市疾病预防控制中心业务综合楼已通过竣工验收；汕尾海关技术业务用房已建成投入使用；华师附中汕尾学校已完成征地工作；汕尾新一代天气雷达项目、101工程、体育馆、市区防洪（潮）等工程进展顺利；汕尾至遮浪一级公路、省道S242线海丰公平至城东段和公平段改线工程、市城区凤飞山至捷胜公路、遮浪至海丰大湖、海丰大湖至陆丰上英、陆丰甲子至南塘公路等有较大进展；厦深铁路（汕尾段）前期工作扎实推进，将于4月份动工建设。列入粤东规划重大项目投资完成情况良好，我市共有建设项目58个，目前已开工建设的有 29个，完成投资42.80亿元。规划、国土工作得到进一步加强，市区市政道路、园林绿化等公共基础设施建设不断推进，城市面貌和环境有了新的提升。

3、"三农"工作迈出新步伐。2007年完成粮食种植面积163.5万亩，增长1.2%,总产54.0万吨，增长2.8%，完成省下达的粮食四项考评指标任务。目前，全市有农业产业化经营组织135家，其中，省、市、县农业龙头企业39 家。渔业（水产品）总产量62.9万吨，渔业经济总产值64.20亿元。林业生态建设、造林绿化、森林资源保护管理以及国有林场建设取得新成绩。扶贫工作稳步推进，劳务输出不断增加。支农惠农政策进一步落实，社会主义新农村建设取得新成就。

4、县域经济获得新成效。海丰县、市城区生产总值分别增长18.1%和20.0%，高于全市平均增长速度。陆丰市固定资产投资达到56.95亿元，增长43.6%。6个县（市、区）财政一般预算收入增幅均在30%以上。各地专业镇、中心镇建设进一步加快。

5、招商引资创出新业绩。全市新批准外商投资项目58个，增长18.4%；合同利用外资4.02亿美元，增长42.3%；实际利用外资2.03亿美元，增长39.8%。外贸进出口总值21.90亿美元，增长41.5%。其中，外贸进口10.67亿美元，增长53.8%；外贸出口11.23亿美元，增长31.5%。信利、德昌等电子集团企业增资扩产达1亿美元。与珠三角等地区的经济协作进一步加强。

6、财税工作实现新跨越。全市地方财政一般预算收入达12.16亿元，比上年增加2.89亿元，增长31.2%，年增量为建市20年最大，财政收支基本平衡。其中，国税部门完成地方收入2.01亿元，增长36.4 %；地税部门完成地方收入5.00亿元，增长27.6%。金融形势稳定，年末金融机构存款余额190.29亿元，比年初增长15.8%，贷款余额70.66亿元，增长8.4%。

7、旅游商贸突显新亮点。创建"中国优秀旅游城市"活动全面展开。旅游配套设施建设稳步推进，目前新建、扩建旅游项目10个。旅游品牌塑造得到加强，全市旅游总收入17.03亿元，增长8.5%，旅游创汇521.2万美元，增长8.7%。全市完成房地产开发建设投资7.32亿元，增长228.7%，实现商品房销售额5.52亿元，增长299.8%。个体私营经济进一步发展。全市现有个体工商户4.8万户，私营企业2280家，实现民营经济增加值175.19亿元，占全市生产总值比重59.3%。全市民营企业获国家"免检产品"和省级名牌、名商标的有21个。

8、和谐汕尾建设呈现新面貌。2007年全市新增城镇就业3.4万人，转移农村劳动力就业4.9万人。城镇失业率控制在3.0%以内。养老、失业、工伤等社会保险覆盖面继续扩大。全市征收五项社保费3.53 亿元，增长16.7%,企业离退休人员养老金等社保待遇按时足额发放,农村合作医疗参合率达78.3%。县（市、区）困难企业军转干部生活困难补贴和驻汕市区部队生活补贴得到提高，城镇退役士兵得到妥善安置，发放抚恤事业费4270万元，重点优抚对象的基本生活得到保障。发放城乡低保保障金4732万元，享受低保救助对象达3.3万户，7.3万人。投入资金2400万元，完成农村危房改造3750户。涉及人民群众切身利益的住房、饮水、打官司、子女读书等问题得到较好解决。环保工作得到加强，重污染行业实行定点管理，污染减排及污染源普查工作扎实推进，全市生态环境总体良好。产品质量及食品安全的监督管理得到强化，市场秩序良好，市民消费安全。

社会管理和维稳工作进一步加强。社会治安综合治理目标管理责任制得到落实，"平安工程"建设扎实推进。各项严打专项整治活动深入开展，"两抢一盗"、凶杀贩毒等违法犯罪活动得到严厉打击。加强对东洲地区的综合整治，汕尾发电厂配套工程竣工投入使用。生产安全事故四项指标全面下降，有效遏制重特大事故的发生。打击走私贩私工作取得成效。公共安全突发事件预防应急管理工作得到强化，应急工作机制进一步完善落实。矛盾排查调处工作不断加强，来信来访持续下降。"行风热线"工作深入开展，热点难点问题得到妥善解决。社会和谐稳定，人民安居乐业，是全省最安全的地区之一。

9、社会事业结出新成果。精神文明建设扎实开展。"社会主义荣辱观"、"爱国、守法、诚信、知礼"和"文明村镇、文明社区、文明行业"等主题活动，以及"五五普法"教育活动深入开展，广大干部群众的整体素质不断增强。

科技创新和人才工作得到加强。电子信息、海洋、农业等技术合作以及传统支柱工业技改取得新成果。人事人才信息网不断完善，人才引进工作继续推进。

教育文化体育卫生工作取得新成效。农村义务教育危房校舍改造工作全面完成，47万多平方米的危房校舍得到改造。农民子女和城镇低保家庭子女实现免费义务教育。普及高中阶段教育稳步推进，教育教学质量不断提高。2007年高考入围人数近万人，创历史新高，首次摘取两个单科满分的全省状元。"欢乐广场、唱响汕尾"广场文化活动持续开展。一批优秀文化作品获省表彰。文化出版市场整治得到加强。广播电视工作取得新进展。全民健身运动以及"迎奥运"活动蓬勃开展。体育场地设施进一步改善。公共卫生三大体系建设以及疾病防治工作取得新成效，农村卫生服务体系建设以及镇村两级服务能力得到加强。新型农村合作医疗制度进一步完善。食品药品放心工程建设有序推进。

其他各项事业蓬勃发展。城乡计划生育工作薄弱环节得到加强。社会救助、救灾救济、残疾人事业不断推进。殡葬管理进一步规范。军政、军民更加团结，双拥模范城创建工作扎实开展。妇女儿童发展规划进一步落实，未成年人和妇女权益得到维护。国防工作进一步加强。统计、审计、人防、气象、防震、台务、物价、司法、港澳、老龄、民族宗教、外事侨务、档案修志、工会、共青团等工作取得新成绩。

10、民主法制和政府自身建设开创新局面。坚持向人大及其常委会报告工作和向政协通报情况制度，自觉接受法律监督和民主监督。人民代表、政协委员和民主党派、海内外各界人士参政议政作用得到较好发挥。基层民主建设和村民自治工作得到加强。行政执法责任制与责任追究制进一步落实，政务公开和政务信息化建设全面推进。有效实施市政府领导及部门领导接听群众电话和接访群众制度。法制宣传、法律援助、人民调解工作得到加强。反腐败斗争和治理商业贿赂深入开展，勤政廉政建设稳步推进，政府自身建设不断加强，办事效率和服务水平进一步提高。

各位代表，2007年取得的成绩，是在省委、省政府和市委的正确领导下，全市人民团结奋斗的结果。在此，我谨代表市人民政府，向全市广大工人、农民、知识分子、干部职工，向驻汕人民解放军、武警官兵、人民警察和各民主党派、人民团体、社会各界人士，表示崇高的敬意！向所有关心支持我市发展的香港和澳门特别行政区同胞、台湾同胞、海外侨胞以及国际友人表示衷心的感谢！

回顾2007年的工作,我们深深体会到，必须做到"五个强化":必须强化发展理念，以科学发展观统领全局，狠抓发展第一要务不放松，坚持科学调控，加快转变经济发展方式，不断优化经济结构，促进经济社会各个环节协调发展，促进区域、城乡协调发展，促进人与自然和谐发展，推动全市经济又好又快发展；必须强化发展氛围，团结广大干部群众，齐心协力，形成加快发展的强大合力。要按照科学执政、民主执政、依法执政的要求，努力营造干事创业的良好风气，改进领导班子思想作风，最大限度地调动全市人民的积极性、主动性和创造性，确保各项工作任务落实到位；必须强化发展措施，全面实施"三基地三经济带"和"八大工程"的战略部署，突出抓好招商引资工作，大力发展县域经济和民营经济，认真抓好生产力骨干项目、基础设施重点项目建设，努力推进经济跨越发展；必须强化发展根基，坚持以人为本，加快推进以改善民生为重点的社会建设，落实稳定第一责任，全力维护社会稳定，为跨越发展提供良好的社会环境；必须强化发展保障，按照"为民、务实、清廉"的要求，深化改革，开拓创新，恪尽职守，全面加强政府自身建设，不断提高施政能力和服务水平。

在肯定成绩的同时，我们也清醒地看到，我市经济社会发展仍存在一些问题和困难，主要是：经济发展基础薄弱、规模小，人均GDP与全省平均水平差距较大；转变经济发展方式任务艰巨；部分重点项目推进缓慢；城市的辐射带动力较弱；自主创新能力比较弱；县域经济发展不均衡，农民渔民人均收入低；财政调控能力不强，社会管理、公共服务、就业和社会保障还存在薄弱环节；社会治安、勤政廉政建设等还有待加强。我们必须高度重视，切实采取措施认真解决。

2008年工作安排

　　2008年是贯彻落实党的十七大精神的重要一年，也是落实粤东会议"三年打基础、五年大变化、十年大发展"和市五次党代会战略部署的关键之年，又是我国改革开放30周年和汕尾建市20周年。因此，做好今年各项工作意义重大、责任重大。从国际形势看，世界经济形势总体向好，新一轮产业转移加快，我国依然是国际资本和产业转移的重要地区；从国内环境看，我国正处于新一轮经济增长周期，实现经济又好又快发展是主流，有利于我市转变经济发展方式，提高经济运行质量；从省内形势看，全省经济继续保持健康快速发展，省委、省政府不断加大对欠发达地区支持力度，为我市的发展提供了前所未有的历史机遇；从我市自身条件看，近年来我市各项工作扎实推进，经济快速发展，主要经济指标增幅排在全省前列，社会局面稳中趋好，社会各项事业全面发展，广大干部群众对建设好汕尾有着强烈的愿望和满腔的热忱。可以说，我市已进入了跨越发展的起步时期。

当前，我们要切实把思想和行动统一到省委的决策部署和省委十届二次全会、市委五届三次全会精神上来，团结带领全市人民继续解放思想，坚持改革开放，迅速掀起解放思想的学习讨论热潮，以解放思想为先导，以科学发展为主题，教育引导各级和广大干部群众紧紧抓住解放思想这个总开关，弘扬与时俱进的精神，顺应全市人民过上更加美好生活的新期待，重视改善民生和发挥人民群众的主动性、创造性，创新发展理念、转变发展方式、破解发展难题、提高发展质量，推动政府工作不断开创新局面，以新一轮思想大解放推动新一轮大发展，让全市人民共享发展成果。

2008年政府工作的总体要求是：高举中国特色社会主义伟大旗帜，以邓小平理论和"三个代表"重要思想为指导，努力实践科学发展观，深入贯彻落实党的十七大、省十次党代会、省委十届二次全会和市五次党代会精神，继续解放思想，坚持改革开放，围绕"三年打基础、五年大变化、十年大发展，尽快赶上全省总体发展水平"的总目标，落实发展第一要务，稳定第一责任，民生第一选择，群众满意第一标准，全面推动科学发展，更加重视改善民生，全力促进社会和谐，加快推进"三基地三经济带"和"八大工程"建设，加快转变经济发展方式，加快完善社会主义市场经济体制，推动产业结构优化升级，提高经济增长质量，大力推进社会事业建设，加快推进城乡协调发展，努力把汕尾建设成为工业、旅游业和海洋产业发达的现代化滨海新城。

今年经济社会发展的主要预期目标是：地区生产总值增长18%左右；农业总产值增长5%；工业总产值增长29%，规模以上工业产值增长40%；第三产业增加值增长15%；社会消费品零售总额增长18%；全社会固定资产投资增长25%；居民消费价格涨幅控制在3%左右；地方财政一般预算收入增长25%；外商直接投资增长25%；外贸出口总额增长20%；城镇登记失业率控制在3%以内；农民人均纯收入增长6%，城镇居民可支配收入增长9%；人口自然增长率控制在5.82&permil;以内；万元GDP能耗控制在0.522/吨标煤以内。

为此，2008年的工作要突出"十个着力":

（一）着力抓好"三大基地"建设，增强经济实力

打造电子信息产业基地。进一步完善和实施电子信息产业基地发展规划，建设好市区核心产业园区，制定并落实有关优惠政策，大力扶持电子信息产业的发展。以信利、德昌电子等骨干企业为龙头，建设电子信息产业基地。大力推进电子信息产业集群招商，重点吸引国内外知名大公司前来投资合作。整合技术、人才、资金、政策、服务等要素，加强产学研合作，把我市信息产业融入泛珠三角电子信息产业带，形成互相配套、协调发展的产业链，促使电子信息产业成为我市的工业支柱产业。

打造电力能源产业基地。抓紧推进汕尾发电厂3、4号机组尽快上马；加快推进陆丰核电站、海丰核电站、陆丰甲湖、甲东风电场等项目建设，加大推进电源输出工程和电网改造工程的力度，尽快把我市建设成为全省乃至全国重要的电力生产基地。

打造石油化工产业基地。大力推进海丰小漠华城储运和生产基地的建设，抓紧做好规划、岸线利用、环评等工作，及专用码头的建设，确保一期工程按期投产，加快推进二、三期工程建设。做好陆丰石化工业园区项目的前期工作，争取国家有关部门的支持。配合好粤东成品油管道（汕尾段）建设，积极引进石油化工中下游产品，大力发展石化工业，打造我市石油化工产业基地。

（二）着力抓好"三大经济带"建设，强化区域协调发展

发展蓝色产业经济带。加大渔港基础设施建设力度，大力发展海洋捕捞业，重点发展水产养殖业和水产品深加工业，加大发展海淡水养殖力度，积极开发应用推广养殖新技术，扶持发展一批省、市级渔业龙头企业，加快水产养殖基地化建设，建立一批高产、优质、高效的养殖基地。加强水生野生动物的保护管理以及贝类等海产资源保护区的管理，加快人工鱼礁区的建设。加强渔船渔场渔港和水产品市场的管理，完善水产品质量安全管理体系。以品清湖的保护利用和整治为重点，开展海域整治，科学保护和利用海域。

发展绿色产业经济带。在确保粮食生产稳步发展的同时，加快发展效益农业、现代农业和特色农业，提高农业市场化、专业化、规模化水平。培育发展一批国家、省、市级农业龙头企业和专业合作社组织，巩固壮大我市4家省级农业龙头企业，扶持培育五丰水产食品有限公司等5家企业为省级农业龙头企业。全面推广"公司+基地+农户"经营方式，不断增加农民收入。加快市农业现代化示范区建设，提升全市农业现代化水平。完善农产品标准体系、质量检测体系和动植物防疫体系建设，切实加强农产品质量安全管理。

发展特色旅游产业经济带。以创建中国优秀旅游城市为契机，大力发展旅游休闲度假业。完善旅游规划，整合旅游资源，以"红、蓝、绿、古"四色旅游为主题，加强星级酒店等旅游基础设施建设，丰富旅游文化内涵，制定精品旅游线路，提升旅游服务质量，打响特色旅游品牌，培育一批有较强竞争力的旅游业龙头企业。争取年底前进入中国优秀旅游城市行列。

（三）着力抓好基础设施建设，夯实发展基础

加快交通基础设施建设。重点抓好汕尾至遮浪一级公路、国道G324线汕尾路段路面大修和改线工程、市城区凤飞山至捷胜公路、省道S242线海丰公平至城东段改线工程、省道S338线甲子至南塘二级公路改造工程、甲子至东港公路等建设；抓紧镇通镇和镇通建制村路面硬底化公路建设。加快汕尾新港区的规划建设，抓紧完善汕尾发电厂专用码头和海丰小漠石化码头建设，加快渔港及渔业加工基地建设。认真配合做好广西玉林至福建漳州高速公路以及汕尾至江西寻乌高速公路汕尾路段的工可及设计工作，全面动工建设厦深铁路（汕尾段）。

加快城市基础设施建设。实施城市"东扩"战略，加快推进市区建设。加快完成全市土地利用总体规划的修编，及市区重点发展地区的控制性详细规划的编制，推动品清湖沿岸综合开发项目建设；严格执行土地利用年度计划，确保重点建设项目的实施；加快市区道路建设步伐，推进市区东片污水处理厂、城市医疗废物处理厂、市区小伯坑垃圾处理场等市政设施建设，抓紧规划建设体育场、展览馆、文化馆等公共活动设施，增强城市服务功能。加强城市管理，大力整治脏乱差，加快城市及其周边地区的植树造林工作，改善城市环境，提升城市品位。

加快水利基础设施建设。全力实施城乡水利防灾减灾工程建设，不断增强全市水利工程的防灾减灾抗灾能力。加快城市防洪（潮）工程建设步伐，进一步完善防洪工程体系和安全保障体系。推进农田水利设施建设，改善农业生产条件。切实加强水资源的合理开发和综合利用。继续实施省人大小型水库除险加固、机电排灌改造、水库移民安置三大议案工程。加快水利管理体制改革，加强全市蓄水工程管理。

（四）着力抓好外源型经济发展，提高利用外资质量

加大招商引资力度。继续实行招商引资领导分工责任制，创新招商引资方式，拓宽招商引资渠道，优化利用外资结构，切实提高利用外资质量和水平。加大对外推介和宣传，认真组织参加各项境内外招商洽谈会。着力引进大财团、大企业到我市投资置业，力争吸收外资取得新突破。

扩大外贸出口。加快转变外贸增长方式，调整进出口结构，促进加工贸易转型升级，大力发展服务贸易。鼓励民营企业发展加工贸易，积极稳妥推进加工贸易企业扩大内销。加强口岸建设，推进临港工业、物流业、保税加工区的建设。努力扩大外贸进出口规模。

优化投资环境。继续推进"大通关"建设，不断改善投资营商环境。提高服务质量，完善优惠政策，推进市行政服务中心建设，进一步改善项目审批和服务，建立集中联合审批制度，为投资者提供优质高效的服务。加强对外经济技术合作与交流，鼓励和支持有条件的企业开展对外投资，发展境外加工贸易、远洋捕捞和劳务输出，推进企业跨国经营。

抓好园区建设。对现有工业园区进行科学规划和优化整合，高效、合理利用土地资源，把建设工业园区和发展产业集群结合起来，构筑工业发展大平台。重点抓好红海湾经济开发试验区、陆丰东海经济开发试验区、海丰老区经济开发试验区、陆丰星都经济开发区等省级开发区的建设。加强与深圳等珠三角地区的合作交流，认真做好与东莞（大朗）、深圳（水田）、中山（沙溪）共建产业转移园区的工作，加强产业转移园区招商，促进在产业、资金、资源、人才、劳动力等方面的优势互补，实现"双赢"。

（五）着力抓好内源型经济发展，做大经济总量

加快重点项目建设。认真落实重点项目领导分工责任制。2008年初步确定市重点建设项目22个，总投资223.48亿元，年度计划投资30.95亿元。重点抓好电子信息、电力能源、石油化工产业以及厦深铁路（汕尾段）、环品清湖沿岸堤路、华师附中汕尾学校和碧桂园、万福等重点项目的建设。继续抓好我市列入省粤东重大产业布局的58个重点项目建设。加强项目的申报和储备，争取多一些项目进入国家、省的计划安排。

做强做大民营经济。落实和完善鼓励扶持民营经济发展的政策措施，实施中小企业成长工程，重点扶持一批"专、精、特、新"的中小企业和民营企业，帮助企业搞好生产经营。拓宽中小企业融资渠道，支持信用担保机构建设。引导民营经济向科技型、外向型、规模效益型方向发展，推动民营经济发展壮大。

增强自主创新能力。加快建立以企业为主体、产学研结合、产品创新为核心的技术创新体系。大力发展高新技术产业，积极申请建立广东汕尾高新技术产业开发区。坚持不懈地运用高新技术和先进适用技术改造提升海洋生物、纺织服装、食品饮料、首饰工艺品和圣诞礼品等传统产业。全面深化产学研合作，以我市龙头企业为依托，与高校、科研院所和上、下游产业企业建立产学研战略联盟，增强自主创新能力，提升产业竞争力。抓好公平、可塘、碣石、东海、东坑等省级技术创新试点镇和公平镇产业集群升级示范区的技术创新。努力推进"火
